# Qualitative analysis of the structural, thermal and rheological properties of a plant ice cream based on soy and sesame milks

**DOI:** 10.1002/fsn3.2037

**Published:** 2021-01-09

**Authors:** Sajad Ghaderi, Mostafa Mazaheri Tehrani, Mohammad Ali Hesarinejad

**Affiliations:** ^1^ Department of Nutrition Faculty of Health and Nutrition Sciences Yasuj University of Medical Science (YUMS) Iran; ^2^ Department of Food Science and Technology College of Agriculture Ferdowsi University of Mashhad Iran; ^3^ Department of Food Processing Research Institute of Food Science and Technology (RIFST) Mashhad Iran

**Keywords:** ice cream, optimization, sesame, soy

## Abstract

The aim of this study was to investigate the structural, physicochemical, and rheological properties of soy and sesame milk‐based ice cream in order to optimize its formula. The overrun percentage of the optimized ice cream was acceptable compared to the conventional ice cream (produced with cow milk). The hardness and consistency of the optimized ice cream were significantly (*p* < .05) higher than those of the conventional one, while its cohesiveness was lower. All the samples showed the pseudoplastic behavior and the power‐law model had a high efficiency (*R* ^2^ ≥ .99) in describing their rheological behavior. The lowest span value, the lowest mean particle diameter, and the highest mean particle surface area, and thus, the most stable and homogenous samples were associated with the conventional ice cream followed by the optimized plant one. The number of air bubbles in the structure of the conventional and optimized ice creams was significantly (*p* < .05) higher than in the other samples. The soy ice cream had the lowest T_g_ (−58.04°C), whereas the conventional one had the highest T_g_ (−55.05°C). Unlike the plant‐based samples, especially the soy ice cream, the conventional ice cream had the lowest ice content (IC), the highest unfreezable water (UFW), and the lowest frozen water (FW). Overall, this ice cream was more acceptable in terms of sensory attributes in comparison with the control sample and could be supplied to consumers as a novel, high‐quality, and marketable ice cream.

## Graphical abstract

The optimized ice cream based on soy and sesame milks have more acceptable in terms of sensory evaluation and other physical properties in comparison with the control sample and could be supplied to consumers as a novel, high quality, and marketable ice cream.

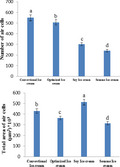

**Research highlights**

- The optimized plant‐based ice cream was produced by the most appropriate ratio of soy and sesame milk on textural parameters
- The textural and sensory properties of optimized ice cream were acceptable
- The high content of plant proteins in the plant‐based ice cream produced causes functional properties
- The optimized plant ice cream could be supplied to consumers as a novel, high‐quality, and marketable ice cream

## 1 INTRODUCTION

Ice cream is a frozen foam comprised of partially aggregated lipid particles, air cells, ice crystals, and a continuous aqueous phase (serum) in which components such as polysaccharides, proteins, lactose, and mineral salts are dispersed (Bahramparvar et al., 2011). Application of plant‐based milk, as the sources which provide plant proteins and lipids in ice cream, not only incorporates the nutritional values and health‐promoting effects of plant compounds into ice cream but also does lead to the production of a novel product with specific properties such as lactose‐free products, which could be attractive and useful for consumers (Quasem et al., 2009). Plant‐based milk is free of cholesterol and has unsaturated fats which increase the health of the cardiovascular system. These products are good alternatives to animal milk and are digested quickly. Some people, such as vegetarians or the ones who are allergic to lactose or casein, have entirely eliminated this dairy product from their diet. Soy milk is a protein solution that contains fat, protein, essential amino acids, minerals, and vitamins. It reduces heart diseases due to their unsaturated fatty acids and lack of cholesterol. On the other hand, by using this milk, the problem of lactose absorption will be resolved (Liu & Lin, 2000). Friedeck et al. (2003) also stated that adding soy protein isolate to ice cream formulation enhanced the herb flavors. Also, Dervisoglu et al. (2005) showed that an increase in soy protein concentrate increased the viscosity and consistency coefficient of samples at all tested temperatures. Therefore, these unpleasant flavors can be improved with other plant milk. One of the sesame's products is an extract termed sesame milk. Sesame milk fat is composed mainly of unsaturated fatty acids. The lack of lactose in this milk is a considerable point; as a result, the problems of absorption of lactose, which are common in dairy products, will be resolved. A very interesting point about sesame milk is its sesame flavor which is desirable unlike other plant‐based flavors and is preferred to soy milk in this respect (Quasem et al., 2009). Some researchers have reported that sesame proteins could be employed as a factor for the preparation of foam, emulsion, and gel in a variety of food products (Escamilla‐Silva et al., 2003).

The demand for animal origin proteins is expected to double by 2050; therefore, it is necessary to look for alternative protein sources that have nutritional and functional properties close to cow's milk. Soybean protein is a promising alternative to animal proteins due to its high nutritional content, good techno‐functional properties, and acceptable cost (Alves & Tavares, 2019). In general, owing to their much larger molecular size and structural restriction through disulfide crosslinks, plant proteins form a comparatively thicker interfacial layer at oil/water interfaces compared to dairy proteins, which can also stabilize multiphase structures such as ice cream (Le Roux et al., 2020).

Owing to its multiphase nature, ice cream needs a strong protein structure. Therefore, to produce plant‐based ice cream with desirable textural properties and sensory attributes, we have to use a mix of plant‐based milk based on soy with a lot of strong protein and sesame with a suitable flavor coating. Therefore, according to all the mentioned cases, the main purpose of this research is to prepare optimal plant ice cream based on soy milk and sesame milk, which is comparable to ordinary ice cream in terms of textural, thermal, structural, and rheological distribution parameters as well as particle size.

## 2 MATERIALS AND METHODS

### 2.1 Materials

Soy flour (19% fat and 16.5% solid nonfat (SNF)) was purchased from Tus Suyan Co. (Mashhad, Iran). Sterilized and homogenized milk (1.5% fat), skimmed milk powder, and homogenized and sterilized cream (30% fat) were supplied from Pegah Dairy Co (Mashhad, Iran). Panisol ex (commercial stabilizer) was provided form Danisco, Demark. Sugar and vanilla were all purchased from local market. High‐quality peeled sesame was obtained from Simorgh Tahini Halva Co. (Mashhad, Iran). All other chemicals used were of analytical grade unless otherwise specified.

### 2.2 Methods

#### 2.2.1 Soymilk preparation

Soy flour and distilled water (1:5) were mixed in a blender at a rate of 700 rpm for 15 min at 85°C. The resulting mixture was then cooled down to 40–50°C and filtered using a two‐layered filter cloth (the final dry matter and protein content of the soymilk was adjusted to 11%–12% and 4.5%, respectively). Finally, it was cooled off and pasteurized at 80°C for 15 s and kept in sealed bags at 4–5°C until use (Yeganehzad et al., 2009).

#### 2.2.2 Sesame milk preparation

Sesame was mixed with distilled water at a ratio of 1:3. The mixture was kept at ambient temperature for 16 hr. Next, the sesame was rinsed with water and after mixing with water at a ratio of 1:2 and was blanched for 15 min at 85°C. After draining, the sesame and distilled water (1:5) were blended in a blender at a rate of 700 rpm for 20 min. After keeping at room temperature for 1 hr, the resulting mixture was filtered using a filter cloth so that the final dry weight of the sesame milk was adjusted to 11%–12% (the final protein content of the sesame milk was 1.8%). Eventually, the prepared milk was pasteurized in suitable containers at 80°C for 15 s and stored in sealed bags at 4–5°C for further experiments (Ahmadian‐Kouchaksaraei et al., 2014).

#### 2.2.3 Preparation of the best ratio of soy milk to sesame milk

The D‐optimal mixture design was used to obtain the optimal ratio of soy milk: sesame milk in the formula of the plant ice cream by using three textural parameters, including hardness, consistency, and apparent elasticity modulus. In the mixture experiment design, the total amount of milk is held constant and the measured property of the samples changes when the proportions of the milk types are changed (0%–100%). All predicted models have been produced starting with full cubic models. Some of the terms have been omitted in order to achieve the most optimal model. In the cubic models, the higher‐order interaction terms with the highest *p* value were gradually eliminated until all model terms were significant (*p* < .05). The models were applied to terms with *p* values smaller than .10 to obtain a more accurate prediction with a higher *R* ^2^ value.

#### 2.2.4 Ice cream preparation

The conventional ice cream was composed of 10% fat, 15% sugar, 11% skimmed milk powder, 0.5% stabilizer, and 0.1% vanilla. A three‐unknown equation was developed in Excel 2010 according to the formula above in addition to the dry matter and fat contents of each of the plant milk and the cow`s milk individually. The mass of all ingredients was determined based on this equation. After weighting all of the required ingredients for the three different formulas of soy, sesame, and conventional ice creams, the liquid substances, including each of the plant milk, cow`s milk, and cream, were stirred continuously upon heating moderately up to 45–50°C. Afterward, the mixture of the solid matter, including sugar, skimmed milk powder, emulsifier, and vanilla, was added to the heated liquid. After being dissolved, they were completely blended using a stirrer (Sunny, Model SM‐65, Germany) for 3 min and pasteurized at 80°C for 25 s and homogenized using a homogenizer (Ultra Turrax T25D IKA, Germany) at 15,000 rpm for 3 min. After that, the mix was immediately cooled down to 5°C using a bath of water, ice, and salt. Then, aging was performed at 5 ± 0.5°C for 24 hr. The prepared ice cream mix was placed in a batch ice cream maker (Model ICK 5000, Delonghi, Germany) during freezing for 20 min. Eventually, the samples were poured into plastic containers, coded, and stored at −18°C for at least 24 hr. Cow's milk was used with the same material and methods for the production of the conventional ice cream.

### 2.3 Experiments

#### 2.3.1 Physicochemical analyses of milk

The pH, total solid, protein, and fat contents were analyzed by the reference AOAC (2000) method and ISO 20483:2013 ISO, 2013. Casein content in the samples was analyzed based on ISO 17997–1:2004 ISO, 2004. According to the pycnometer method, the pycnometer was firstly weighed empty, and then, it was filled once by distilled water and after that by the ice cream mix at 25°C and weighed each time. The specific gravity (density relative to water) of the mix was determined by the following equation:

(1) $$\text{Specific}\;\text{gravity}=\frac{G_{3}‐G_{1}}{G_{2}‐G_{1}}$$

where G1, G2, and G3 are the weights of the empty pycnometer, the pycnometer plus distilled water, and the pycnometer plus ice cream mix, respectively (Marshal & Arbukle, 1996).

Overrun was measured by the following equation:

(2) $$\text{Overrun}\;\left(\%\right)=\frac{\text{weight}\;\text{of}\;\text{ice}\;\text{cream}\;\text{mix}‐\text{weight}\;\text{of}\;\text{ice}\;\text{cream}}{\text{weight}\;\text{of}\;\text{ice}\;\text{cream}}\times 100$$

#### 2.3.2 Rheological analysis

After 24 hr of aging, the apparent viscosity of the ice cream mixes was determined using a rotational viscometer (Bohlin Model Visco 88, Bohlin instruments, UK) equipped with a thermal circulator (Julabo, Model F12‐MC, Julabo Labortechnik, Germany) at a shear rat of 51.8 s^−1^ at 5 ± 0.5°C. The empirical data were fitted to the power–law model (Akalın et al., 2008).

#### 2.3.3 Particle size analysis

Mean diameter, particle size distribution, and specific surface area (SSA) were measured by using a particle size analyzer (particle size analysts 22, Germany) through the dynamic light scattering (DLS) method. This device measures the mean geometric diameter of the particles in addition to the width of the particle size distribution curve, called Span. For this purpose, the ice cream samples were kept at 4°C for 4 hr after the hardening step during 24 hr at −18°C. They were then diluted and homogenized with distilled water at a ratio of 1:1,000. The samples were stored in a refrigerator at a temperature of 4°C until the test was completed. The samples were completely homogenized before the analysis.

#### 2.3.4 Textural analysis

After the 50 g samples of the plant‐based and conventional ice creams were kept at ambient temperature for 5 min, a texture analyzer (Brookfield CT3‐10kg, the USA) was employed to analyze their texture. To that end, a cylinder probe, 6 mm in diameter, was chosen to penetrate 15 mm into the sample at a rate of 2 mm/s (Akalın et al., 2008).

#### 2.3.5 Thermal analysis

In order to evaluate the thermal properties of the ice cream samples, differential scanning calorimetry (DSC) was used after the aging process (DSC 822, Switzerland). The analysis was performed according to Soukoulis et al. (2009). The thermal cycles were applied as follows: (a) Cooling to −60°C at 10°C/min. (b) Heating from −60°C to 10°C at 10°C/min. (c) Cooling from 10°C to −60ºC at 10°C/min.

#### 2.3.6 Optical microscopy

An optical microscope (Olympus BX51, Japan) equipped with a camera mounted on it (Olympus BP71, Japan) was used to investigate the samples structure. For image acquisition, a certain amount of each sample (0.2–0.5 mg) was compressed between two glass slides after 24h of slow hardening. Then, 10 images were captured using the 40 μm lens to acquire the highest quality image. Subsequently, ImageJ 1.44P software was used to analyze the images (Chang & Hartel, 2002).

#### 2.3.7 Sensory evaluation

After 24 hr of storage at −18°C, the sensory evaluation of the ice cream samples was performed by 10 trained panelists through the 9‐point hedonic scale. The investigated attributes included flavor, taste, texture, color, appearance, and overall acceptability (Herald et al., 2008).

#### 2.3.8 Statistical analysis

Design Expert software version 10, D‐optimal mixture design were applied to optimize the formula, and the best ratio of soy milk to sesame milk was selected. Then, this study was also conducted based on a completely randomized design using SPSS software version 20. All analyses were performed in triplicate from the same bulk of sample. Duncan's multiple range test was exploited for mean comparison at 95% confidence interval (*p* < .05), and the graphs were drawn using Microsoft Office Excel 2010. Different letters were used to indicate values which were significantly different.

## 3 RESULTS AND DISCUSSION

### 3.1 Formula optimization based on textural parameters

Formula optimization was performed based on three interconnected textural parameters, including hardness, consistency, and modulus of elasticity. Five soy milk: sesame milk ratios were considered. Based on the experimental results, the best proportion of soy milk (55%) to sesame milk (45%) with a low *p* values was obtained for the preparation of the plant‐based ice cream with the best texture characteristics. To validate the optimal formula proposed by the software, an experiment was conducted in duplicate in which the optimal ratio of the milk was used to prepare ice cream and the measured responses were compared with the predicted results. As shown in Table 1, the experimental and predicted results were very close to each other, indicating the validity of the model and the values obtained for the ratio between the two plant milk.

**Table 1:** Average values of experimental and predicted results (validity test)

Treatment	Experimental results	Predicted results	*R* ^2^	*MSE*
Hardness	1,217.91	1,209	.9697	0.0085
Consistency	122.6	111.7	.9540	0.0122
Apparent elasticity modulus	196.39	195.42	.9613	0.0032

### 3.2 Physicochemical properties

The results of this study (Table 2) showed that pH did not show any significant differences (*p* < .05), because the percentages of the nonfat solids of the milk and the fat content of the formula were constant. There have been no significant differences among the pH values of different ice cream samples in similar studies (Aykan et al., 2008; Karaca et al., 2009). The protein and casein values of the samples were also calculated due to the importance of this compound in the development of functional characteristics in ice cream. The highest amount of protein was found in soy ice cream, followed by the optimized ice cream and the lowest amount of protein was related to the conventional ice cream.

**Table 2:** Comparison between the physicochemical, rheological, and particle size indices of the ice cream samples

Sample	Physicochemical properties		Rheological properties				Particle size analysis				
	pH	Protein content (casein)	Apparent viscosity (Pa.s)	Flow index (*n*)	Consistency coefficient (*k*)	*R* ^2^	Arithm Mean Diameter (µm)	Uniformity	Mean/Median	specific surface area (Sqr (m)/cc)	Span
Soy ice cream	6.8 ± 0.24^a^	3.35 (0.82) ± 0.41^a^	1.0245 ± 0.03^a^	0.619 ± 0.01^bc^	4.63 ± 0.04^a^	.99	4.18^a^	1.09^a^	1.61^a^	3.24^c^	3.14^a^
Sesame I ce cream	6.59 ± 0.13^a^	2.88 (1.68) ± 0.41^b^	0.18452 ± 0.03^c^	0.742 ± 0.04^a^	0.506 ± 0.02^d^	.99	3.74^b^	0.88^b^	1.45^ab^	3.55^c^	2.76^b^
Conventional ice cream	6.72 ± 0.11^a^	2.73 (2.73) ± 0.38^b^	0.29187 ± 0.09^b^	0.672 ± 0.03^b^	1.059 ± 0.09^b^	.99	2.18^c^	0.92^b^	1.51^a^	4.17^b^	2.39^c^
Optimized ice cream	6.89 ± 0.16^a^	3.07 (1.1) ± 0.64^ab^	0.43556 ± 0.07^b^	0.637 ± 0.02^bc^	1.795 ± 0.07^c^	.99	2.04^c^	0.49^c^	1.13^b^	5.49^a^	1.54^d^

Note

Different letters in each column indicate significant differences (*p* < .05).

**Figure 1:**
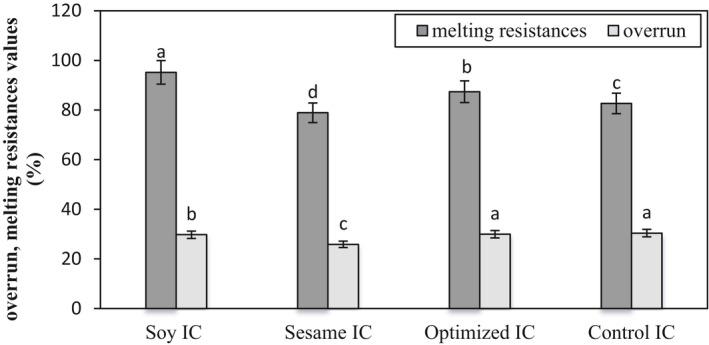
Comparison of the overrun and melting resistances values of the ice cream samples. *Different letters on the columns show significant differences (*p* < .05)

The overrun percentage of the optimized plant‐based ice cream did not have a significant difference (*p* < .05) compared to the conventional ice cream sample, as its overrun value was almost equal to that of the conventional ice cream (Figure 1). Also, the percentages of resistance to melting of all the ice cream samples (Figure 1) were significantly different (*p* < .05) from each other. The percentage of resistance to melting of the optimized ice cream was significantly (*p* < .05) less than that of the soy ice cream, and more than those of the conventional and sesame ice creams. Given that the type and amount of all effective non‐protein compounds, including stabilizers, dry matter, and fat, as well as the method of ice cream production, were similar in all samples; this result seems to be justified considering the difference in the amount and type of soy protein and casein in samples (their functional role, especially high water absorption). Shahrabi et al. (2011) stated that soy proteins had the ability to make foam and can play an effective role in bulky food products. Mahdian and Mazaheri (2011) investigated the effect of whole soy flour on the characteristics of ice cream. Their results showed that a rise in the substitution level of nonfat solids with soy flour up to 45% increased the volume of the samples. It can be stated that hydrophilic functional groups released more free water in the form of hydration water and by decreasing free water, increased the micro‐viscosity in the nonfrozen phase (Serum) of the ice cream, and thus increased resistance to melt (Damodaran et al., 2007).

### 3.3 Rheological properties

The results of the rheological behavior showed that the power‐law model had a high efficiency (*R* ^2^ = .99) to describe the rheological behavior of the plant ice cream mixes (Table 2). The results also demonstrated that the viscosity and consistency coefficient of the soy and optimized ice cream mixes were largely influenced by the use of soy proteins and were significantly (*p* < .05) higher than those of the conventional and sesame ones (Table 2). Therefore, it can be said that all of the mechanisms affecting the increase in viscosity and emulsion stability influenced the melting resistance of the ice creams. It can also be claimed that the main reason for the higher viscosity and consistency coefficient was the presence of proteins and polysaccharides of soy milk in the ice cream, because the presence of these compounds with high molecular weight, through mixing with water and forming a gel network, can justify the viscosity (Bahramparvar et al., 2008). Dervisoglu et al. (2005) and Herald et al. (2008) achieved similar results. According to the results in addition to *n* ≤ 1 for all the samples, it would appear that all the samples had a pseudoplastic behavior, which is consistent with the findings of Marshall et al. (2003) and Soukoulis et al. (2009).

### 3.4 Particle size distribution

The most important variable in the analysis of the particle shapes is the width of the diagrams, named Span. The narrower width of the shape, the smaller the span and consequently, the greater the uniformity, and stability of the mixture. According to results, the narrowest width of the shape, the lowest span value and thus the most stability and homogeneity were related to the conventional ice cream samples followed by the optimized ice cream (Table 2). On the other hand, the widest range of shape and the highest value of span and consequently, the least stability were associated with the soy ice cream sample. The dispersity index of the particle size of a system is quite effective on the system stability; as a result, with increasing particle size dispersity, mass transfer occurs from small to large particles resulting in the instability of the system. Consequently, in any formula where the Span index is lower, the stability of the system will be greater.

The results of the average diameter of the particles and the ratio of the diameter to the mean of the particles (Table 2) showed that the conventional ice cream followed by the optimized one had the smallest particle size and the diameter to mean ratio. At the same time, the soy ice cream had the highest average diameter and the diameter to mean ratio among all the samples; therefore, these results clearly showed that the conventional ice cream was the most stable and homogeneous mixture and the optimized ice cream, compared to the other plant ones, had the most homogeneous mixture and the closest results to the conventional ice cream.

In the case of SSA (Table 2), it can be stated that the smaller the particle size, the greater its surface area. Consequently, in this study, the largest level was assigned to the conventional ice cream with the smallest particle size followed by the optimized one. With regard to the functional characteristics and the higher level of casein activity and the proteins present in soy milk and sesame seeds, it can be said that this protein, with more superficial activity at and possibly through the formation of effective complexes with emulsifiers and other hydrocolloids in the formula, reduced the particle size, increased the SSA, and homogenized the mixture of the conventional and optimized ice creams. These results are justifiable in case that Bolliger et al. (2000) cited that the total protein content of the ice cream mixture increased the surface area of the particles and stabilized the ice cream level. They also mentioned that the effect of emulsifiers was very important on key variables in the ice cream mixture. The decrease and increase in the size of the fat particles and the instability of the measured particle size depend entirely on the amount of functional proteins in the ice cream mixture. Mohamed et al. (2005) declared that active materials, with effective placement between surfaces reduced the effective area of the particles and thus reduced the particle size, so the more active and functional the compounds of the structure, the smaller the particles.

### 3.5 Textural properties

Since the size of the ice crystals and the ice‐free phase volume are involved in creating hard texture in ice cream, this factor can be considered to be a criterion for the growth of ice crystals (Muse & Hartel, 2004). The hardness and consistency of the sesame ice cream (Table 3) were significantly higher than those of the other samples (*p* < .05). On the other hand, the hardness and consistency of the optimized ice cream were significantly higher than those of the conventional one (*p* < .05). It can be stated that the amount of the optimized ice cream hardness was closer to that of the conventional ice cream. Definitely, the change in the type and amount of protein and fat in the plant and cow's milk for the preparation of various samples had a significant effect on the functional and important properties of ice cream, which influenced the size and distribution of air bubbles and the formation of ice cream crystals, and could ultimately change the texture hardness and consistency of the ice cream. Hardness may be considered a reflection of the mixture components (fat, protein, sugars, and hydrocolloids) and the process conditions (homogenization, aging, and freezing) of the final product (Varela et al., 2014). Sesame ice cream adhesiveness was significantly (*p* < .05) higher than that of the other samples, and the adhesiveness of the optimized sample was very similar to that of the conventional ice cream. This parameter depends on the combined effect of adhesiveness and bonding forces in addition to other factors such as viscosity and viscoelasticity (Adhikari et al., 2001). The results (Table 3) also showed that the apparent elasticity modulus of the optimized plant‐based ice cream was significantly (*p* < .05) more than that of the other ice cream samples.

**Table 3:** Comparison between the textural and thermal of the ice cream samples

Sample	Textural properties				Thermal properties					
	Hardness (g)	Adhesiveness (mj)	Apparent elasticity modulus (kPa)	Consistency (mj)	Glass Transition Temperature (°C)	Latent Heat of Melting (J/g)	UFW (%)	FW (%)	IC (%)	Freezing point (°C)
Soy ice cream	1,442 ± 4.35^b^	4.2 ± 0.73^c^	125.0347 ± 1.35^b^	128.1 ± 2.38^b^	−58.04	−141.70	20.99	79.01	42.41	−0.88
Sesame ice cream	1,820 ± 6.65^a^	8.7 ± 0.98^a^	110.174 ± 1.28^c^	158.9 ± 2.81^a^	−56.55	−126.95	25.41	74.59	37.99	−1.13
Conventional ice cream	836 ± 3.18^d^	6.9 ± 0.72^b^	89.744 ± 2.34^d^	72.5 ± 1.17^d^	−57.30	−139.81	21.55	78.45	41.84	−0.92
Optimized ice cream	1,209 ± 2.11^c^	4.4 ± 0.69^c^	195.42 ± 2.63^a^	111.7 ± 2.29^c^	−55.05	−114.68	29.07	70.92	34.32	−1.39

Note

Different letters in each column indicate significant differences (*p* < .05).

### 3.6 Thermal properties

T'g is one of the most important characteristics of ice cream that is effective on its thermodynamic stability during storage. In fact, this temperature is a very important variable in terms of thermodynamic stability during storage, formulation of food products, and designing food processes (Heldman et al., 2018; Soukoulis et al., 2009). As the results of this study show, the soy ice cream showed the lowest T_g_ (−58.04°C) and the conventional one had the highest T_g_ (−55.5°C). These results seem to be directly related to the amount and type of protein present in the system so that the proteins of the system can maintain water molecules and therefore reduce the movement of these molecules, resulting in less water in the system and finally has a direct positive effect on phase change and T_g_. By increasing the amount of casein proteins present in the system (due to its high functional power), T_g_ increased (Table 3).

The results demonstrated that the conventional and soy ice cream samples had the lowest and highest freezing points, respectively (Table 3). Goff and Hartel (2013) and Soukoulis et al. (2009) maintained that nonfat milk solids (minerals and lactose) and sweeteners (monosaccharaides and disaccharides) directly contributed to the degradation of freezing point, and they are the main factors for reducing the freezing point and, on the other hand, protein, fat, high molecular weight carbohydrates, stabilizers, and emulsifiers were indirectly involved in the degradation of the freezing point; therefore, one of the main reasons for this drop of freezing point is the presence of lactose in the conventional ice cream compared with the plant‐based ones. On the other hand, the results of the melting curve showed that the conventional ice cream had the lowest enthalpy and, in fact, the lowest freezing point. On the other hand, the soy ice cream had the highest enthalpy and the highest freezing point indicating that the most uniform crystals belonged to the conventional ice cream and the soy ice cream had nonuniform crystals. This can be attributed to the higher potential of the conventional ice cream protein in free water bonding. The results of this study are consistent with those of Mehditabar et al. (2015) who stated that by increasing the level of MSNF replacement in ice cream with solid squash puree, the total protein content of the final mixture was reduced, thereby reducing the glass transition temperature because of a decrease in the majority of protein; therefore, the addition of squash puree in this study reduced the thermodynamic stability of the ice cream. Also, they stated that adding pumpkin nuts to ice cream generally reduced the ice volume, increased the amount of nonfreezing water, and reduced the frozen water.

In contrast to the plant‐based ice creams, the conventional ice cream had the lowest amount of ice, the highest volume of UFW and the lowest FW (Table 3). The most important reason for this positive phenomenon can be attributed to the presence of the compounds that can bind strongly with water such as proteins (especially casein) and the lower freezing point of the ice cream. Alvarez et al. (2005) mentioned that improving the sensory properties of ice cream would depend on the increased homogeneity of the size distribution of ice crystal. This creates a narrow range of melting temperature. Knowing the amount of ice for understanding the ice cream behavior during freezing and analyzing the physical structure and morphology of frozen ice cream is very important (Cogné et al., 2003). Hwang et al. (2009) found out that the main reason for the reduction of enthalpy in the ice cream containing grape wine was the reduction in the amount of frozen water.

### 3.7 Microscopic properties

**Figure 2:**
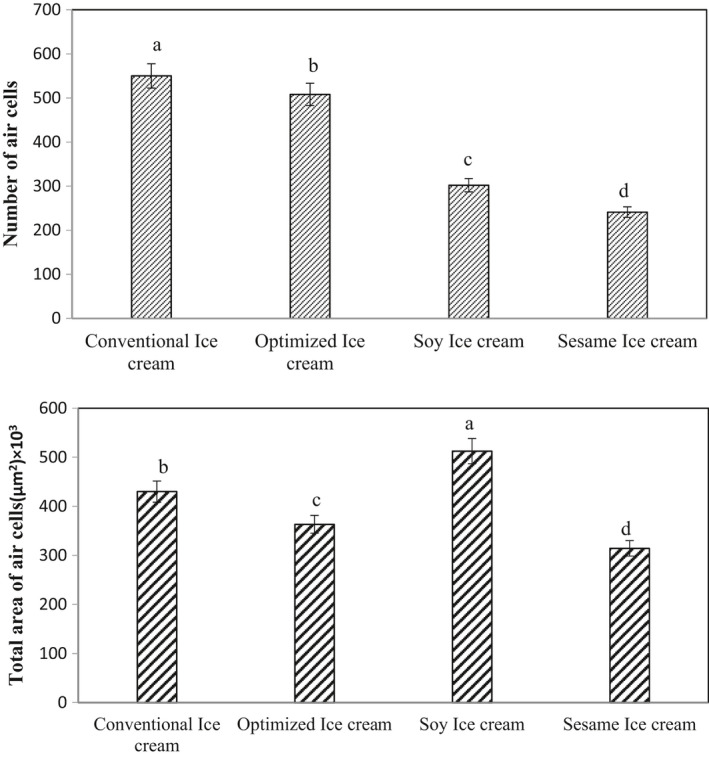
The number and total area of air bubbles in the ice cream samples. *Different letters on columns show significant differences (*p* < .05)

Sofjan and Hartel (2004) evaluated the role of air volume on ice cream properties and stated that the larger the amount of air in the ice cream and the more uniform its distribution, the less firm the ice cream, the higher its overrun and the more resistant it is to melting. The analysis of the images of ice cream samples showed that the number of air bubbles in the conventional ice cream followed by the optimized one was significantly (*p* < .05) higher than in the other samples (Figure 2). On the other hand, an examination of the total area of air bubbles in the ice cream (Figure 2) revealed that the soy ice cream followed by the conventional one had the highest volume of air bubbles and the smallest.

Dervisoglu et al. (2005) reported that surface active agents such as milk proteins, emulsifiers, and stabilizers improved the mixing properties and reduced the size of air bubbles. They also stated that the volume of the foam depended on the protein concentration. Soy proteins have the potential to compete with milk proteins in terms of functional properties, so a larger and more significant amount of soy protein supplements can increase the volume of air bubbles in this ice cream. At the same time, the small number of air bubbles in this ice cream can justify the weakness of the functional and active properties of soy proteins in comparison with casein. In a way that conventional ice cream with the highest amount of casein had the most uniform and the largest number of air bubbles, which is why it had the highest overrun and lowest texture hardness among the samples (Figure 1 and Table 3). In the case of the optimized ice cream, it can be said that this ice cream had desirable results in terms of the number and volume of air bubbles due to the proper amount of plant proteins and the presence of casein and its results were favorable in comparison with the conventional ice cream. In the case of the sesame ice cream, despite the amount and type of the desired proteins, there were no acceptable results for the number and volume of air bubbles, which may be due to the weakness of the functional properties of the proteins in sesame milk and, on the other hand, a fundamental change in the structure of fat was found in the formula. Due to the small amount and volume of bubbles in the sesame ice cream, the highest hardness belonged to this sample. Razavi et al. (2001) also found similar results, consistent with those of this study, on the effect of soy proteins on the number and volume of air bubbles.

### 3.8 Sensory evaluation

**Figure 3:**
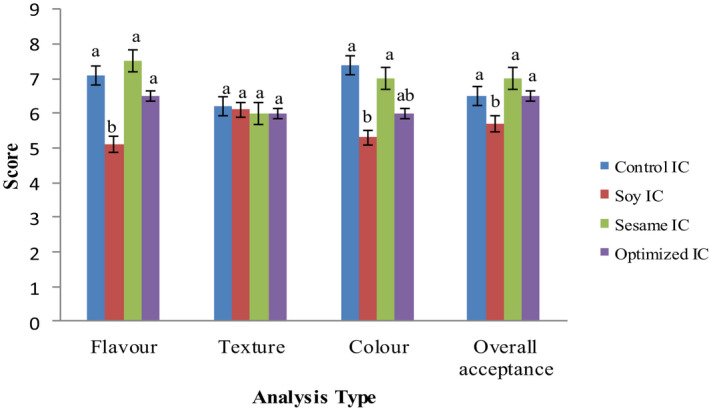
Sensory evaluation of ice cream samples. *Different letters on each column indicate significant difference (*p* < .05)

The results of sensory evaluation showed that (Figure 3) the plant‐based ice cream samples were generally good for production, and only the soy ice cream was not acceptable in terms of taste (*p* < .05) because of the use of soy milk which is justifiable. Nevertheless, in the case of the other sensory attributes, the scores showed that there was not a significant difference (*p* > .05) between among the samples in terms of texture, so the points were very close to each other.

## 4 CONCLUSION

The results of this study showed that although there were significant differences in the physicochemical, textural, rheological, and thermal properties of the ice cream samples, the optimized plant‐based ice cream was close to the conventional one and, in some cases, even had a better quality. High quality and content of plant proteins were due to formation of functional properties in optimized ice cream sample. The overrun percentage of the optimized and conventional ice cream were close together (29.9, 30.4).The hardness (1,209, 836) and consistency (111, 72) of the optimized ice cream were significantly higher than those of the conventional one (*p* < .05). Investigation of the PSA curves show that the most stable samples were the conventional and optimized ice creams, respectively, (span [1.54, 2.39] and mean particle diameter [2.04, 2.18]). Also, the soy and conventional ice cream had the lowest T_g_ (−58.04°C) and the highest T_g_ (−55.05°C), respectively. The results of sensory evaluation also confirmed the quality and general acceptance of the plant‐based ice cream. According to these results, new products with unique characteristics can be produced which eliminate the healthy problems involved in many animal products, including the presence of cholesterol, and replace animal proteins and fats with the plant‐based ones.

## CONFLICT OF INTEREST

The authors confirm that there is no known conflict of interest associated with this publication. This article does not contain any studies with human participants or animals performed by any of the authors. Written informed consent was obtained from all study participants.

## Data Availability

The data that support the findings of this study are available from the corresponding author upon reasonable request.

## References

[fsn32037-bib-0001] Adhikari, B. , Howes, T. , Bhandari, B. R. , & Truong, V. (2001). Stickiness in foods: A review of mechanisms and test methods. International Journal of Food Properties, 4(1), 1–33. 10.1081/JFP-100002186

[fsn32037-bib-0002] Ahmadian‐Kouchaksaraei, Z. , Varidi, M. , Varidi, M. J. , & Pourazarang, H. (2014). Influence of processing conditions on the physicochemical and sensory properties of sesame milk: A novel nutritional beverage. LWT‐Food Science and Technology, 57(1), 299–305. 10.1016/j.lwt.2013.12.028

[fsn32037-bib-0003] Akalın, A. S. , Karagözlü, C. , & Ünal, G. (2008). Rheological properties of reduced‐fat and low‐fat ice cream containing whey protein isolate and inulin. European Food Research and Technology, 227(3), 889–895. 10.1007/s00217-007-0800-z

[fsn32037-bib-0004] Alvarez, V. B. , Wolters, C. L. , Vodovotz, Y. , & Ji, T. (2005). Physical properties of ice cream containing milk protein concentrates. Journal of Dairy Science, 88(3), 862–871. 10.3168/jds.S0022-0302(05)72752-1 15738219

[fsn32037-bib-0005] Alves, A. C. , & Tavares, G. M. (2019). Mixing animal and plant proteins: Is this a way to improve protein techno‐430 functionalities? Food Hydrocolloids, 97, 105171.

[fsn32037-bib-0006] AOAC . (2000). Official methods of analysis of the association of official analytical chemists, Vol. 9. Association of Official Analytical Chemists.

[fsn32037-bib-0007] Aykan, V. , Sezgin, E. , & Guzel‐Seydim, Z. B. (2008). Use of fat replacers in the production of reduced‐calorie vanilla ice cream. European Journal of Lipid Science and Technology, 110(6), 516–520. 10.1002/ejlt.200700277

[fsn32037-bib-0008] Bahramparvar, M. , Hadad Khodaparast, M. H. , & Mohamad, A. A. (2008). Effect of substitution of carboxymethylcellulose and salep gums with Lallemantia royleana hydrocolloid on ice cream properties. Iranian Food Science and Technology Research Journal, 4(1), 37–47.

[fsn32037-bib-0009] Bahramparvar, M. , & Mazaheri, T. M. (2011). Application and functions of stabilizers in ice cream. Food Reviews International, 27(4), 389–407. 10.1080/87559129.2011.563399

[fsn32037-bib-0010] Bolliger, S. , Goff, H. D. , & Tharp, B. W. (2000). Correlation between colloidal properties of ice cream mix and ice cream. International Dairy Journal, 10(4), 303–309. 10.1016/S0958-6946(00)00044-3

[fsn32037-bib-0011] Chang, Y. , & Hartel, R. W. (2002). Development of air cells in a batch ice cream freezer. Journal of Food Engineering, 55(1), 71–78. 10.1016/S0260-8774(01)00243-6

[fsn32037-bib-0012] Cogné, C. , Andrieu, J. , Laurent, P. , Besson, A. , & Nocquet, J. (2003). Experimental data and modelling of thermal properties of ice creams. Journal of Food Engineering, 58(4), 331–341. 10.1016/S0260-8774(02)00396-5

[fsn32037-bib-0013] Damodaran, S. , Parkin, K. L. , & Fennema, O. R. (2007). Fennema's food chemistry. CRC Press.

[fsn32037-bib-0014] Dervisoglu, M. , Yazici, F. , & Aydemir, O. (2005). The effect of soy protein concentrate addition on the physical, chemical, and sensory properties of strawberry flavored ice cream. European Food Research and Technology, 221(3–4), 466–470. 10.1007/s00217-005-1207-3

[fsn32037-bib-0015] Escamilla‐Silva, E. M. , Guzmán‐Maldonado, S. H. , Cano‐Medinal, A. , & González‐Alatorre, G. (2003). Simplified process for the production of sesame protein concentrate. Differential scanning calorimetry and nutritional, physicochemical and functional properties. Journal of the Science of Food and Agriculture, 83(9), 972–979.

[fsn32037-bib-0016] Friedeck, K. G. , Karagul‐Yuceer, Y. , & Drake, M. A. (2003). Soy protein fortification of a low fat dairy‐based ice cream. Journal of Food Science, 68(9), 2651–2657.

[fsn32037-bib-0017] Goff, H. D. , & Hartel, R. W. (2013). Ice cream. Springer Science & Business Media.

[fsn32037-bib-0018] Heldman, D. R. , Lund, D. B. , & Sabliov, C. (Eds.) (2018). Handbook of food engineering. CRC Press.

[fsn32037-bib-0019] Herald, T. J. , Aramouni, F. M. , & Abu‐Ghoush, M. H. (2008). Comparison study of egg yolks and egg alternatives in French Vanilla ice cream. Journal of Texture Studies, 39(3), 284–295. 10.1111/j.1745-4603.2008.00143.x

[fsn32037-bib-0020] Hwang, J. Y. , Shyu, Y. S. , & Hsu, C. K. (2009). Grape wine lees improves the rheological and adds antioxidant properties to ice cream. LWT‐Food Science and Technology, 42(1), 312–318. 10.1016/j.lwt.2008.03.008

[fsn32037-bib-0021] ISO . (2004). Milk—Determination of the casein‐nitrogen content—Part 1: Indirect method (Reference method). ISO 17997–1. International Organization for Standardization (ISO), Geneva, Switzerland.

[fsn32037-bib-0022] ISO 20483 . (2013). Cereals and pulses ‐ Determination of the nitrogen content and calculation of the crude protein content – kjeldahl method. ISO.

[fsn32037-bib-0023] Karaca, O. B. , Guven, M. , Yasar, K. , Kaya, S. , & Kahyaoglu, T. (2009). The functional, rheological and sensory characteristics of ice creams with various fat replacers. International Journal of Dairy Technology, 62(1), 93–99.

[fsn32037-bib-0024] Le Roux, L. , Mejean, S. , Chacon, R. , Lopez, C. , Dupont, D. , Deglaire, A. , & Jeantet, R. (2020). Plant proteins partially replacing dairy proteins greatly influence infant formula functionalities. Food Science and Technology., 120, 108891.

[fsn32037-bib-0025] Liu, J. R. , & Lin, C. W. (2000). Production of kefir from soymilk with or without added glucose, lactose, or sucrose. Journal of Food Science, 65(4), 716–719.

[fsn32037-bib-0026] Mahdian, E. , & Mazaheri, T. M. (2011). Optimization of process condition and formulation of soy‐cow milk mix for probiotic yoghurt ice cream production. Ferdowsi University of Mashhad Faculty of Agriculture. PhD dissertation.

[fsn32037-bib-0027] Marshal, R. T. , & Arbukle, W. S. (1996). Formulas and industry standards: Ice Cream, (Ed.): Arbukle, W. S., (5th ed., pp. 314‐327). Chapman and Hall.

[fsn32037-bib-0028] Marshall, R. T. , Goff, H. D. , & Hartel, R. W. (2003). Composition and properties in Ice Cream, (pp. 11–54). Springer.

[fsn32037-bib-0029] Mehditabar, H. , Razavi, S. M. A. , & Elahi, M. (2015). Effect of pumpkin puree on the physicochemical, rheological, thermal and sensorial properties of ice cream. Ferdowsi University of Mashhad Faculty of Agriculture. MSc Thesis.

[fsn32037-bib-0030] Mohamed, M. A. N. , Ranjard, L. , Catroux, C. , Catroux, G. , & Hartmann, A. (2005). Effect of natamycin on the enumeration, genetic structure and composition of bacterial community isolated from soils and soybean rhizosphere. Journal of Microbiological Methods, 60(1), 31–40. 15567222 10.1016/j.mimet.2004.08.008

[fsn32037-bib-0031] Muse, M. R. , & Hartel, R. W. (2004). Ice cream structural elements that affect melting rate and hardness. Journal of Dairy Science, 87(1), 1–10. 10.3168/jds.S0022-0302(04)73135-5 14765804

[fsn32037-bib-0032] Quasem, J. M. , Mazahreh, A. S. , & Abu‐Alruz, K. (2009). Development of vegetable based milk from decorticated sesame (Sesamum indicum). American Journal of Applied Sciences, 6(5), 888. 10.3844/ajassp.2009.888.896

[fsn32037-bib-0033] Razavi, S. M. A. , Habibi, M. B. , & Nayebzadeh, K. (2001). Effect of dairy substituents and stabilizers on chemical and physical properties of soy ice cream (Parvin). Iranian Journal of Agricultural Sciences, 32(3), 615–624.

[fsn32037-bib-0034] Shahrabi, A. A. , Badii, F. , Ehsani, M. R. , Maftoonazad, N. , & Sarmadizadeh, D. (2011). Functional and thermal properties of chickpea and soy‐protein concentrates and isolates. Iranian Journal of Nutrition Sciences & Food Technology, 6(3), 49–58.

[fsn32037-bib-0035] Sofjan, R. P. , & Hartel, R. W. (2004). Effects of overrun on structural and physical characteristics of ice cream. International Dairy Journal, 14(3), 255–262. 10.1016/j.idairyj.2003.08.005

[fsn32037-bib-0036] Soukoulis, C. , Lebesi, D. , & Tzia, C. (2009). Enrichment of ice cream with dietary fibre: Effects on rheological properties, ice crystallisation and glass transition phenomena. Food Chemistry, 115(2), 665–671. 10.1016/j.foodchem.2008.12.070

[fsn32037-bib-0037] Varela, P. , Pintor, A. , & Fiszman, S. (2014). How hydrocolloids affect the temporal oral perception of ice cream. Food Hydrocolloids, 36, 220–228. 10.1016/j.foodhyd.2013.10.005

[fsn32037-bib-0038] Yeganehzad, S. , Tehrani, M. M. , Shahidi, F. , & Zayerzadeh, E. (2009). Study on the effect of soymilk on survival of Lactobacillus acidophilus, physicochemical and organoleptical properties of probiotic yoghurt. Journal of Agricultural Sciences and Natural.

